# Wave interference at the contralateral ear helps explain non-monotonic envelope interaural time differences as a function of azimuth

**DOI:** 10.1121/10.0017631

**Published:** 2023-03-23

**Authors:** Paul G. Mayo, Andrew D. Brown, Matthew J. Goupell

**Affiliations:** 1 Department of Hearing and Speech Sciences, University of Maryland, College Park, Maryland 20742, USA; 2 Department of Speech and Hearing Sciences, University of Washington, Seattle, Washington 98105, USA paulmayo@umd.edu, andrewdb@uw.edu, goupell@umd.edu

## Abstract

Interaural time differences (ITDs), an important acoustic cue for perceptual sound-source localization, are conventionally modeled as monotonic functions of azimuth. However, recent literature and publicly available databases from binaural manikins demonstrated ITDs conveyed by the envelopes (ENV-ITDs) of high-frequency (≥2 kHz) signals that were non-monotonic functions of azimuth. This study demonstrates using a simple, time-dependent geometric model of an elliptic head that the back-traveling (longer) sound path around the head, delayed and added to the conventionally treated front-traveling path, can account for non-monotonic ENV-ITDs. These findings have implications for spatial-hearing models in acoustic and electric (cochlear-implant) hearing.

## 1. Introduction

Acoustic-hearing listeners use binaural cues—interaural time differences (ITDs) and interaural level differences (ILDs)—for azimuthal-plane sound localization. For acoustic-hearing listeners, there is a dominant role of ITD in the low-frequency (<1.5 kHz) temporal fine-structure of sounds (FS-ITD) and negligible role of ITD in the high-frequency (≥1.5 kHz) fine-structure (Wightman and Kistler, 1992; Macpherson and Middlebrooks, 2002; Klug and Dietz, 2022). Acoustic-hearing listeners are also sensitive to ITD in the temporal envelope (ENV-ITD), particularly when no usable ITD is present in the temporal fine structure (FS-ITDs) (Henning, 1974; Bernstein and Trahiotis, 2002; Bernstein and Trahiotis, 2012). ENV-ITDs are of particular interest to those studying spatial hearing in bilateral cochlear-implant (BiCI) listeners, as clinical cochlear-implant processing typically discards temporal fine structure leaving only the temporal envelope, therefore precluding access to low-frequency FS-ITDs but potentially preserving access to ILDs and ENV-ITDs (Aronoff *et al.*, 2010; Grantham *et al.*, 2008).

The simplest models of interaural differences are primarily geometrical (e.g., the “airhead” model consisting of two point receivers separated by some distance; Hartmann, 2021) and yield a simple monotonic relation in which larger azimuths yield larger cues. (For simplicity, we will only consider binaural cues at 0° through 90° azimuth because of the approximately mirror-symmetrical cues expected in other quadrants.) Physical heads produce complex diffraction effects, yielding frequency-dependent ILDs that are highly non-monotonic functions of azimuth (Kuhn 1977; Mayo and Goupell, 2020) and can result in the mislocalization of tones in acoustic-hearing listeners (Macaulay *et al.*, 2010). FS-ITDs are also frequency-dependent (Kuhn 1977), and are monotonic functions of azimuth (Feddersen *et al.*, 1957; Kuhn 1977). Non-monotonic frequency-dependent ILDs can be reasonably modeled as the scattering of sound by a spherical head (Macaulay *et al.*, 2010). FS-ITDs have also been estimated from static phase differences between antipodal points using spherical head models (Kayser *et al.*, 2009; Middlebrooks and Green, 1990). However, many of these models (at least those that, to our knowledge, are used within the context of binaural hearing) are not defined as a function of time. The temporal dynamics of head-related physics, particularly their interactions with signals such as those with prominent modulation envelopes, are therefore not well-accounted for in conventional models of binaural cue generation.

A recent study examining binaural cue transmission in the context of BiCI processing, and thus evaluating ENV-ITDs in relatively low-frequency regions of the spectrum not typically considered for this binaural cue, reported acoustic ENV-ITDs that varied non-monotonically across azimuth (Gray *et al.*, 2021). This result was surprising, given the expected monotonicity of ITD cues (e.g., Feddersen *et al.,* 1957; Kuhn, 1977). Most notably, sound sources at intermediate azimuths yielded non-monotonic ENV-ITDs for narrowband signals centered at approximately 1.5 kHz [see Gray *et al.* (2021), Fig. 4(C)]. Subsequent evaluation of available head-related transfer function (HRTF) libraries measured using different test fixtures across different test facilities (e.g., Kayser *et al.,* 2009, Gardner and Martin, 1995, and Denk and Kollmeier, 2020) revealed that non-monotonic ENV-ITD-azimuth functions in this spectral region are a reproducible result. The present report attempts to explain this observation using a simple time-dependent model.

For a sound originating within the frontal hemifield and away from the midline, the sound traveling around the front of the head has a shorter distance to the ear farther from the source (the contralateral ear) than the sound traveling around the back of the head. The “back-traveling sound” thus arrives at the contralateral ear later than the front-traveling sound, and this arrival time delay causes interference at the contralateral ear at certain frequencies and azimuths. The consequences of this interference considered in the amplitude spectrum (observed via the head-related transfer function, HRTF) give rise to ILD non-monotonicity, the well-known “acoustical bright spot” phenomenon (e.g., Macaulay *et al.,* 2010). Here, we hypothesized that such interference, considered in a time-dependent fashion, could also affect the temporal envelope of amplitude-modulated sounds, therefore altering ENV-ITDs. To test our hypothesis, we developed a simple geometric model in the time domain to simulate the effect of back-traveling sound paths on observed ENV-ITDs.

## 2. Methods

### 2.1 Acoustic head model

**Fig. 1.**
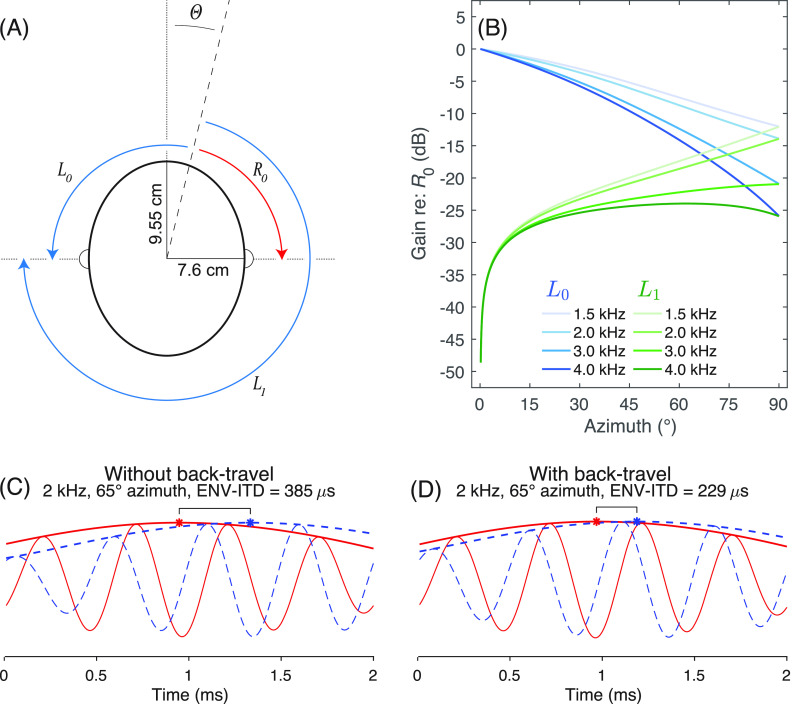
An overview of the acoustic head model. Panel (A) illustrates an ellipse with the dimensions used in this study as well as the front-traveling paths that a sound at source azimuth, θ, takes to reach the right ear ( $R_{0}$) and left ear ( $L_{0}$). It also illustrates the paths that back-traveling sound takes to reach the left ear ( $L_{1}$). Panel (B) shows the accumulated gain relative to $\theta_{R0}$ of each path due to head shadow, incident wave opposition, and the inverse-square law all as a function of azimuth and center frequency. The gain for frequencies <1.5 kHz is equal to that of 1.5 kHz and therefore not shown. Panel (C) shows the left ear (dashed blue line) and right ear (solid red line) waveforms with highlighted temporal envelopes (bold lines) of a 2-kHz bandpass-filtered speech segment originating at 65° azimuth after processing by the model without the back-traveling path. Red and blue asterisks represent respective envelope maxima with a bracket to emphasize the ENV-ITD. Panel (D) is the same as panel (C), but the model includes the back-traveling path. Introducing the back-traveling path shifts the peaks of the left-ear temporal envelope relatively forward in time, effectively reducing ENV-ITD.

Figure 1 shows the results of a model that simulates head-related impulse responses (HRIRs)—the time-domain equivalent of HRTFs—for time-dependent creeping waves around an elliptical head for multiple source azimuths and center frequencies. It accounts for sound traveling around both the front and back of the head and simulates acoustic phenomena that affect level such as the inverse square law, head shadow, and opposition to the incident wave. It then applies the HRIRs to bandpass-filtered speech and calculates the ENV-ITD from the resulting signals.

For a given source azimuth θ ( 0°≤θ≤90°), the angular distance that a creeping wave travels to reach destination x is defined as $\theta_{x}$, where x may be the right ear or left ear reached via either the front- or back-traveling paths. To simulate ears positioned at ±90° azimuth, $\theta_{x}$ is equal to θ−90° for the right ear ( $\theta_{R0}$) and θ+90° for the left ear ( $\theta_{L0}$) via the front-traveling paths. For the back-traveling path, $\theta_{x}$ is equal to 270°−θ ( $\theta_{L1}$) for the left ear. A schematic of the multiple paths to each ear is provided in Fig. 1(A).

The propagation time for a creeping wave to travel around the partial circumference of an ellipse can be calculated using the Incomplete Elliptic Integral of the Second Kind converted to time, here defined as

$$c\left(\theta_{x}\right)=\frac{a}{s}\int_{r=0{}^{\circ}}^{\theta_{x}}\sqrt{1-\left(1-\frac{b^{2}}{a^{2}}\right){\text{sin}\left(r\right)}^{2}}dr,$$ (1)

which returns the arc length from 0° to $\theta_{x}$ for an ellipse with semi-major axis, a, and semi-minor axis, b, and converted to time by dividing by the speed of sound, s, or 343 m/s [Eq. (1)]. In this study, a was 9.55 cm and *b* was 7.6 cm, or half the nominal head length and breadth of head and torso simulators defined in ITU-T recommendation P.58 (ITU-T, 2013).

The equation for the left-ear HRIR, L, consists of a Kronecker impulse, δ, at time sample $t=c(\theta_{L0})$ and an additional attenuated impulse at time $c(\theta_{L1})$. The gain of the first impulse in L was determined using a simplistic model of head shadow,

$$h\left(\theta ,f\right)=1-\frac{\theta }{90{}^{\circ}}+\frac{\theta }{90{}^{\circ}}{\left[\frac{\text{min}\left(2a,\frac{s}{f}\right)}{2a}\right]}^{2},$$ (2)

which returns unity gain for any center frequency, f, with a wavelength longer than the major axis of the ellipse (i.e., the head length or 2 a), and linearly decreasing gain as a function of θ when f has a wavelength shorter than the major axis of the ellipse. For an ellipse with the dimensions used in this report, this leaves frequencies below approximately 1.8 kHz unattenuated. The gain of the second impulse in L was the amount of head shadow at f and θ compounded with an additional stage of sub-linearly increasing gain as a function of θ meant to simulate the level of the back-traveling path to the left ear. All paths also account for the inverse square law relative to the distance the front-traveling paths take to reach the ears at 0° azimuth. The combined equation for L was therefore,

$$\begin{array}{c} L\left[t,\theta ,f\right]=\frac{c{\left(90{}^{\circ}\right)}^{2}}{c{\left(\theta_{L0}\right)}^{2}}\cdot h\left(\theta ,f\right)\cdot \delta \left[t-c\left(\theta_{L0}\right)\right]+\sqrt{\frac{\theta }{90{}^{\circ}}}\cdot \frac{c{\left(90{}^{\circ}\right)}^{2}}{c{\left(\theta_{L1}\right)}^{2}}\cdot h\left(\theta ,f\right)\cdot \delta \left[t-c\left(\theta_{L1}\right)\right], \end{array}$$ (3)

where the first and second terms in the sum correspond to the first and second impulses, respectively.

The equation for the right-ear HRIR, R, consists of simply a unit impulse at time $c\left(\theta_{R0}\right)$. The combined equation for R was therefore,

$$R\left[t,\theta ,f\right]=\delta \left[t-c\left(\theta_{R0}\right)\right],$$ (4)

where the first and second terms in the sum correspond to the first and second impulses, respectively. The levels of $L_{0}$ and $L_{1}$ (relative to $R_{0}$) as a function of azimuth are illustrated for multiple center frequencies in Fig. 1(B). In addition, Fig. 1(C) illustrates stereo waveforms for bandpass-filtered speech with a center frequency of 2 kHz when only front-travel paths (i.e., $L_{0}$ and $R_{0}$) are considered. When the back-travel path is added (i.e., $L_{1}$), the ENV-ITD illustrated by the black bracket in Fig. 1(C) decreases as shown in Fig. 1(D).

### 2.2 Comparison with public HRTF databases

The ENV-ITDs created by the “back-travel” binaural model were compared to those measured from two publicly available HRTF databases (Kayser *et al.* 2009; Gardner and Martin 1995) HRTF databases. The results were also compared to a conventional “front-travel” model, which was identical to the back-travel model but contained only the front-traveling paths (i.e., $L_{0}$ and $R_{0}$). The front-travel model was derived from the back-travel model described above as current models of ITDs (spherical head models; e.g., Hartmann, 2021) are usually defined only for sinusoidal sources and therefore do not represent modulated signals. The HRIRs were convolved with a spoken sentence (“Tight curls get limp on rainy days”) from a speech corpus database (IEEE, 1969) digitally bandpass filtered into bands centered at 0.75, 1, 1.5, 2, 3, and 4 kHz using 6th-order forward-backward Butterworth filters with a bandwidth of 500 Hz. ENV-ITD was then numerically calculated at 0° to 90° azimuth in 0.1° steps for the binaural model and 5° steps for the HRTF databases using the interaural cross-correlation of the Hilbert temporal envelopes of the resulting signals. For comparison, the output of the models was linearly scaled to match the values at 90° to the average of the two HRTF databases at 90°.

## 3. Results

**Fig. 2.**
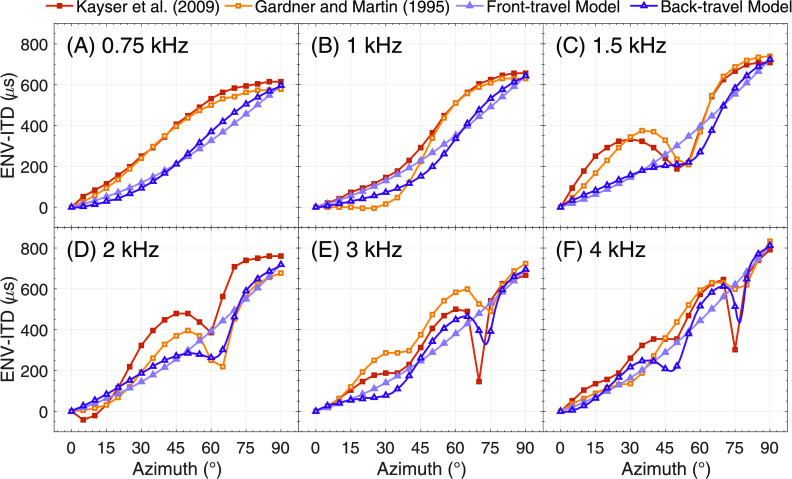
ENV-ITDs as a function of azimuth and center frequency for two publicly available HRTF databases, for a conventional front-travel-only model and for the back-travel model. The different databases/models are denoted by line and each panel represents a different center frequency.

**Fig. 3.**
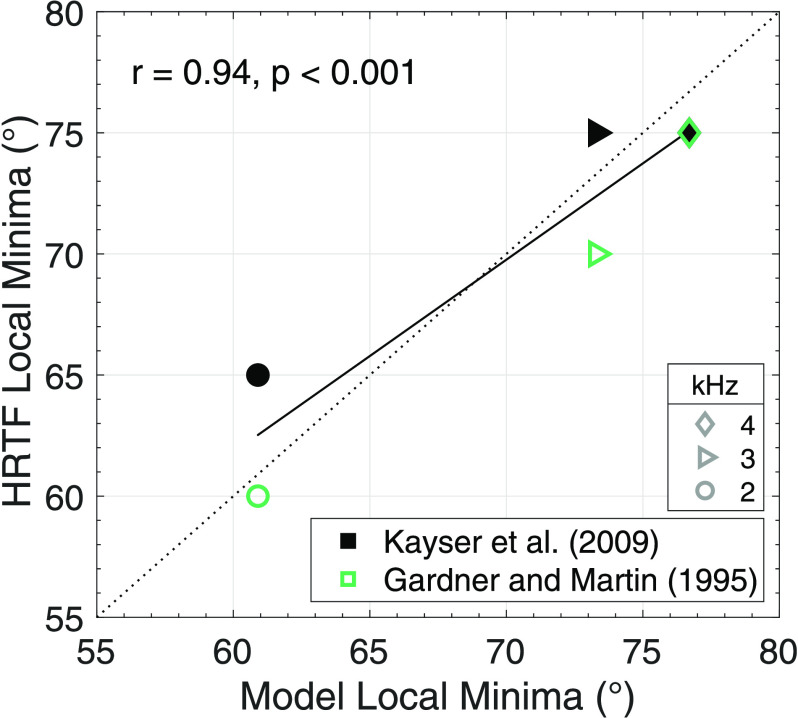
The azimuths corresponding to local minima in the HRTF databases (denoted by symbols) as a function of azimuths corresponding to local minima in the back-travel model. Data are shown for center frequencies where local minima could be identified using the matlab function “*islocalmin*” (denoted by marker shape). Note that at 4 kHz, the open and closed symbols overlap. The dotted line represents a 1-to-1 reference for visual comparison to the data. The solid line represents the line of best fit.

The results are shown in Fig. 2, which shows ENV-ITDs from both models and HRTF databases (denoted by marker shape) as a function of azimuth for each center frequency. All six panels show generally increasing ENV-ITDs as a function of azimuth for all models and HRTF databases. At 0.75 [Fig. 2(A)] and 1 kHz [Fig. 2(B)], for which wavelengths well exceed the simulated head size, ENV-ITDs are generally monotonic in all cases. At 1.5 kHz [Fig. 2(C)], for which the wavelength begins to approach head size, clearly identifiable local minima emerge in the HRTF databases at approximately 55° azimuth. The front- and back-travel models yield monotonic ENV-ITDs, yet a deflection (not deep enough to be a local minimum) appears in the back-travel model near 55° azimuth. For 2, 3, and 4 kHz [Figs. 2(D)–2(F), wavelengths smaller than the simulated head size], ENV-ITD reversals are clearly observed for the HRTF databases although they are at slightly different azimuths and have different depths. These reversals also occur for the back-travel model while the front-travel model remains monotonic. Additionally, the azimuths corresponding to these reversals are similar in each case. The performance of the back-travel model was assessed statistically by regressing the azimuths corresponding to local ENV-ITD minima for the HRTF databases onto those from the back-travel model. Local minima were calculated using the matlab (the MathWorks, Natick, CT) function “*islocalmin”* on the ENV-ITD vs azimuth functions shown in Fig. 2. The results are shown in Fig. 3, which plots the HRTF databases' local minima for frequencies where minima were identified (denoted by marker shape) as a function of the model's local minima. The resulting relationship was nearly 1-to-1 (represented by the dotted line for comparison) and resulted in a statistically significant correlation (*r* = 0.94, *p* < 0.001; solid line in Fig. 3).

## 4. Discussion

The goal of this report was to further document the non-monotonic ENV-ITD-azimuth functions observed in previous work (e.g., Gray *et al.,* 2021) and evident in public HRTF databases (e.g., Gardner and Martin, 1995; Kayser *et al.,* 2009), and offer a potential explanation for this phenomenon. Using a primarily geometric elliptic head model with a simple time-dependency (Fig. 1), the results of this paper suggest that for sounds originating off the midline, sound traveling around the back of the head gives rise to non-monotonicities in ENV-ITD-azimuth functions (Fig. 2). Furthermore, these non-monotonicities arise at similar frequencies and azimuths as those observed in binaural mannequin measurements (Fig. 3), with comparable results observable in other HRTF databases as well (data not shown; e.g., Denk and Kollmeier, 2020). These findings imply that while there appears to be little discussion of non-monotonic ENV-ITD-azimuth functions in the literature (to our knowledge, only one other study has reported them; Macaulay *et al.,* 2017), such non-monotonicities evidently occur and could potentially impact perception. Furthermore, they imply conventional time-independent models of binaural cue generation may fail to capture more complex and time-dependent interactions of real signals propagated around the head.

There are many possible explanations for the minimal prior discussion of non-monotonic ENV-ITD-azimuth functions. One explanation is that previous findings may have been disregarded as errors since ITDs are conventionally held to be monotonic. Another explanation is that there are simply fewer studies concerning ENV-ITDs than FS-ITDs and ILDs. For acoustic-hearing listeners, ENV-ITDs (excepting signal onsets) are less perceptually salient than other cues (Macpherson and Middlebrooks, 2002). However, ENV-ITDs are one of the two binaural cues accessible to BiCI listeners (the other being ILDs). Considering ILDs are highly non-monotonic functions of azimuth, ENV-ITDs may be construed as a potentially more systematic binaural cue available to BiCI listeners, and many studies have focused on prospects for improving sensitivity to ENV-ITD in the BiCI population. The present results suggest that, apart from device and auditory factors that limit access to ENV-ITDs, fundamental acoustic factors may constrain the utility of the cue. Although the present data were generated from models and in-the-ear microphones of binaural mannequins (i.e., the signals that an acoustic-hearing listener would receive), similar results were obtained using data recorded from BiCI microphones (data not shown). This is an expected result since the non-monotonicity arises from the multi-path sound field around the head, thus persisting even when “ear” position is adjusted.

The present results suggest that new models for binaural cues as a function of azimuth that are defined as a function of *frequency and time* could improve model accuracy and benefit the field of binaural hearing. Many of the existing models of azimuthal-plane ITDs, including those derived from analytic solutions like spherical scattering, are not defined as a function of time (Kuhn, 1977; Duda and Martens, 1998). Additionally, these models are often only defined for a sinusoidal point source, and therefore analysis of multi-frequency modulated signals like those used here that would generate an ENV-ITD is not realizable. Extension of the many analytic and numerical approximations of acoustic and electromagnetic scattering with spherical and cylindrical interferers that are defined as a function of time (e.g., Harrington, 2001) could prove fruitful.

The present approach was intended to test the simple hypothesis that time-dependency, and particularly the contribution of delayed back-traveling sound, could account for non-monotonic ENV-ITD functions of azimuth. The geometric model was thus also simple, and it is limited in several aspects. For example, human pinnae are slightly posterior on the head, and therefore simulating ear angles greater than ±90° would be a reasonable improvement (Aaronson and Hartmann, 2014). The model also did not consider source distance or include facial prominences (e.g., a nose). This better highlighted the cause and effect of back-traveling sound on ENV-ITD non-monotonicity while minimizing possible confounds but does not capture the complex geometry of real heads. Additionally, while simulating an elliptic head is more anatomically accurate than a circular head, it does not account for out-of-plane paths (i.e., sound traveling over or under the head) or possible higher-order in-plane paths (e.g., sound traveling back toward the ipsilateral ear). In Figs. 2(C)–2(D), there are local maxima in the HRTF ENV-ITD-azimuth functions at approximately 45° azimuth. This local maximum is marginally present in the back-travel model in Fig. 2(D), but is not present at all in Fig. 2(C), one point of discrepancy between the results of the back-travel model and the empirical HRTFs. It is possible that accounting for additional paths could further increase the prominence of these maxima and the accuracy of the model. Our analyses were also limited to a single filter bandwidth and therefore a single maximum modulation rate. Preliminary analyses revealed an effect of modulation rate on ENV-ITD non-monotonicity, with higher rates resulting in less prominent non-monotonicities (data not shown). The analyses also revealed that non-monotonicities occur when using equivalent rectangular bandwidth filters (Moore and Glasberg, 1983), meaning the observed non-monotonicities are likely present after peripheral processing in acoustic hearing. Investigating the effects of modulation rate, signal bandwidth, and temporal signal features will be a worthwhile endeavor for future research, particularly in addition to how those signal factors may affect the perceptual consequences of these non-monotonicities.

## References

[1] Aaronson, N. L. , and Hartmann, W. M. (2014). “ Testing, correcting, and extending the Woodworth model for interaural time difference,” J. Acoust. Soc. Am. 135, 817–823. 10.1121/1.4861243 25234890 PMC3985894

[2] Aronoff, J. M. , Yoon, Y.-S. , Freed, D. J. , Vermiglio, A. J. , Pal, I. , and Soli, S. D. (2010). “ The use of interaural time and level difference cues by bilateral cochlear implant users,” J. Acoust. Soc. Am. 127, EL87–EL92. 10.1121/1.3298451 20329812 PMC2833183

[3] Bernstein, L. R. , and Trahiotis, C. (2002). “ Enhancing sensitivity to interaural delays at high frequencies by using ‘transposed stimuli,’ ” J. Acoust. Soc. Am. 112, 1026–1036. 10.1121/1.1497620 12243151

[4] Bernstein, L. R. , and Trahiotis, C. (2012). “ Lateralization produced by interaural temporal and intensitive disparities of high-frequency, raised-sine stimuli: Data and modeling,” J. Acoust. Soc. Am. 131, 409–415. 10.1121/1.3662056 22280602 PMC3283900

[5] Denk, F. , and Kollmeier, B. (2020). “ The Hearpiece database of individual transfer functions of an in-the-ear earpiece for hearing device research,” Acta Acust. 5, 1–16. 10.1051/aacus/2020028

[6] Duda, R. O. , and Martens, W. L. (1998). “ Range dependence of the response of a spherical head model,” J. Acoust. Soc. Am. 104, 3048–3058. 10.1121/1.423886

[7] Feddersen, W. E. , Sandel, T. T. , Teas, D. C. , and Jeffress, L. A. (1957). “ Localization of high‐frequency tones,” J. Acoust. Soc. Am. 29, 988–991. 10.1121/1.1909356

[8] Gardner, W. G. , and Martin, K. D. (1995). “ HRTF measurements of a KEMAR,” J. Acoust. Soc. Am. 97, 3907–3908. 10.1121/1.412407

[9] Grantham, D. W. , Ashmead, D. H. , Ricketts, T. A. , Haynes, D. S. , and Labadie, R. F. (2008). “ Interaural time and level difference thresholds for acoustically presented signals in post-lingually deafened adults fitted with bilateral cochlear implants using CIS+ processing,” Ear Hear. 29, 33–44. 10.1097/aud.0b013e31815d636f 18091105

[10] Gray, W. O. , Mayo, P. G. , Goupell, M. J. , and Brown, A. D. (2021). “ Transmission of binaural cues by bilateral cochlear implants: Examining the impacts of bilaterally independent spectral peak-picking, pulse timing, and compression,” Trends Hear. 25, 1–23. 10.1177/23312165211030411 PMC8785329 34293981

[11] Hartmann, W. M. (2021). “ Localization and lateralization of sound,” in *Binaural Hearing*, edited by R. Y. Litovsky , M. J. Goupell , A. N. Popper , and R. R. Fay ( Springer International Publishing, Cham Switzerland), pp. 9–45.

[12] Harrington, R. F. (2001). “ Cylindrical wave functions,” in *Time-Harmonic Electromagnetic Fields* ( IEEE Press, Piscataway, NJ), Chap. 5, pp. 198–205.

[13] Henning, G. B. (1974). “ Detectability of interaural delay in high‐frequency complex waveforms,” J. Acoust. Soc. Am. 55, 84–90. 10.1121/1.1928135 4815755

[14] Institute of Electrical and Electronics Engineers (1969). “ IEEE recommended practice for speech quality measurements,” IEEE Trans. Audio Electroacoust. 17(3), 225–246. 10.1109/TAU.1969.1162058

[15] ITU-T (2013). “ Head and torso simulator for telephonometry,” in *ITU-T Recommendation* ( International Telecommunications Union, Geneva, Switzerland), p. 58.

[16] Kayser, H. , Ewert, S. D. , Anemüller, J. , Rohdenburg, T. , Hohmann, V. , and Kollmeier, B. (2009). “ Database of multichannel in-ear and behind-the-ear head-related and binaural room impulse responses,” EURASIP J. Adv. Sign. Proc. 2009, 157–110. 10.1155/2009/298605

[17] Klug, J. , and Dietz, M. (2022). “ Frequency dependence of sensitivity to interaural phase differences in pure tones,” J. Acoust. Soc. Am. 152, 3130–3141. 10.1121/10.0015246 36586867

[18] Kuhn, G. F. (1977). “ Model for the interaural time differences in the azimuthal plane,” J. Acoust. Soc. Am. 62, 157–167. 10.1121/1.381498

[19] Macaulay, E. J. , Hartmann, W. M. , and Rakerd, B. (2010). “ The acoustical bright spot and mislocalization of tones by human listeners,” J. Acoust. Soc. Am. 127, 1440–1449. 10.1121/1.3294654 20329844 PMC2856510

[20] Macaulay, E. J. , Rakerd, B. , Andrews, T. J. , and Hartmann, W. M. (2017). “ On the localization of high-frequency, sinusoidally amplitude-modulated tones in free field,” J. Acoust. Soc. Am. 141, 847–863. 10.1121/1.4976047 28253653 PMC6910042

[21] Macpherson, E. A. , and Middlebrooks, J. C. (2002). “ Listener weighting of cues for lateral angle: The duplex theory of sound localization revisited,” J. Acoust. Soc. Am. 111, 2219–2236. 10.1121/1.1471898 12051442

[22] Mayo, P. G. , and Goupell, M. J. (2020). “ Acoustic factors affecting interaural level differences for cochlear-implant users,” J. Acoust. Soc. Am. 147, EL358–EL362. 10.1121/10.0001088 PMC7176459 32359311

[23] Middlebrooks, J. C. , and Green, D. M. (1990). “ Directional dependence of interaural envelope delays,” J. Acoust. Soc. Am. 87, 2149–2162. 10.1121/1.399183 2348020

[24] Moore, B. C. J. , and Glasberg, B. R. (1983). “ Suggested formulae for calculating auditory‐filter bandwidths and excitation patterns,” J. Acoust. Soc. Am. 74, 750–753. 10.1121/1.389861 6630731

[25] Wightman, F. L. , and Kistler, D. J. (1992). “ The dominant role of low‐frequency interaural time differences in sound localization,” J. Acoust. Soc. Am. 91, 1648–1661. 10.1121/1.402445 1564201

